# Cold exposure induced oxidative stress and apoptosis in the myocardium by inhibiting the Nrf2-Keap1 signaling pathway

**DOI:** 10.1186/s12872-018-0748-x

**Published:** 2018-02-15

**Authors:** Peifang Cong, Yunen Liu, Nannan Liu, Yubiao Zhang, Changci Tong, Lin Shi, Xuelei Liu, Xiuyun Shi, Ying Liu, Zhou Tong, Mingxiao Hou

**Affiliations:** Emergency Medicine Department of General Hospital of Shenyang Military Command, Laboratory of Rescue Center of Severe Wound and Trauma PLA, No. 83 Road, Shenhe District, Shenyang, l10016 China

**Keywords:** Cold exposure, Oxidative stress, Apoptosis, Nrf2, Mice

## Abstract

**Background:**

Exposure to cold weather is associated with infaust cardiovascular responses, including myocardial infarction and arrhythmias. However, the exact mechanisms of these adverse changes in the myocardium under cold stress are unknown. This study was designed to investigate the mechanisms of cardiac injury induced by cold stress in mice.

**Methods:**

The mice were randomly divided into three groups, normal control (no handling), 1-week cold stress and 2-week cold stress. We observed physiological changes of the mice and morphological changes of myocardium tissues, and we measured the changes of 3′-nitrotyrosine and 4-hydroxynonenal, the expression levels of superoxide dismutase-1, superoxide dismutase-2, Bax, Bad, Bcl-2, Nuclear factor erythroid-derived 2-like 2 (Nrf2) and Kelch like-ECH-associated protein 1 (Keap1) in myocardium by western blot. Besides, we detected mRNA of superoxide dismutase-1, superoxide dismutase-2, Bax, Bad, Bcl-2, Nrf2 and Keap1 by real-time PCR. One-way analysis of variance, followed by LSD-t test, was used to compare each variable for differences among the groups.

**Results:**

Echocardiography analyses demonstrated left ventricle dysfunction in the groups receiving cold stress. Histological analyses witnessed inflammation, vacuolar and eosinophilic degeneration occurred in left ventricle tissues. Western blotting results showed increased 3′-nitrotyrosine and 4-hydroxynonenal and decreased antioxidant enzymes (superoxide dismutase-1 and superoxide dismutase-2) in the myocardium. Expression of Nrf2 and Keap1 followed a downward trend under cold exposure, as indicated by western blotting and real-time PCR. Expression of anti-apoptotic protein Bcl-2 also showed the same trend. In contrast, expression of pro-apoptotic proteins Bax and Bad followed an upward trend under cold exposure. The results of real-time PCR were consistent with those of western blotting.

**Conclusions:**

These findings were very significant, showing that cold exposure induced cardiac injury by inhibiting the Nrf2-Keap1 signaling pathway.

## Background

Exposure to cold weather is broadly considered to be a global challenge to human health, affecting Europe, the United States and many other countries [1–5]. The highest seasonal death rates occur in winter months and the relationship between an extreme cold climate and increased cardiovascular morbidity and mortality is apparent [2, 6–8]. A retrospective analysis in Canada suggested that a 3.0% increase in non-accidental deaths occurred with each 5 °C increase in daily temperature [8]. A cold climate has an especially adverse effect on cardiovascular morbidity and death among people under age 65 [7, 8]. Many clinical and epidemiological studies confirmed an association of cold exposure with infaust cardiovascular responses, for example, increased blood pressure and viscosity and compromised myocardial hemodynamics and performance, all potentially leading to myocardial infarction and arrhythmias [2, 3, 9–12]. Nevertheless, the exact mechanisms of these adverse changes in the myocardium under cold stress remain unknown.

During environmental stresses, oxidative stress is implicated in cellular activities in inflammatory responses, apoptosis and damage to multiple systems, including in cardiovascular disease [13–16]. Nrf2, a transcription factor, plays a protective role by regulating expression of antioxidant proteins that act against oxidative damage [17]. Nrf2 is co-localized in the cytoplasm with Keap1 and Cullin 3 (Cul3). These promote its rapid degradation by ubiquitination under physiological conditions. However, when the Keap1-Cul3 ubiquitination system is disrupted by oxidative stress, Nrf2 translocates into the nucleus from the cytoplasm to bind to a DNA promoter, the antioxidant response element (ARE), in the upstream region of several antioxidant genes, thus initiating their transcription [18, 19]. However, specific mechanisms involving oxidative stress under cold exposure require further exploration.

## Methods

### Mice and cold exposure model

The study was approved by the Animal Care and Use Committee of the General Hospital of Shenyang Military Command. Thirty 8–10 w old Kunming mice were obtained from Liaoning Provincial Laboratory Animal Public Service Center (China, Benxi city). An equal number of male and female mice were maintained at 22 ± 2 °C for the control group and exposed to low ambient temperature in an artificial cold climate chamber (− 20 °C) for the experimental groups. The experimental protocol involved exposure at − 20 °C for 4 h per day for 1 or 2 w, for the two respective experimental groups. Body weights were the same among groups. All animals were raised in the Animal Facility, supplied with food and water ad libitum under a 12-h light—dark cycle, before assessments of myocardial morphology and function. For these assessments, mice were anesthetized with ketamine (100 mg/kg) and xylazine (10 mg/kg).

### Echocardiographic assessment

Mice were anesthetized and evaluated for cardiac geometry and function by animal echocardiography (Vevo 770, a 12 MHz transducer). During diastole and systole, the anterior and posterior wall dimensions of the left ventricle (LV) were determined from three consecutive cycles. Fractional shortening was defined as the ratio of the duration from LV end-diastolic to end-systolic diameters. Positive and negative LV dP/dt_max_ were measured through the right common carotid artery into the ascending aorta by pressure catheter (Scisense Inc., Canada).

### Histopathological assessment

Cardiac tissues were excised, fixed in 10% formaldehyde buffer at room temperature and embedded in paraffin by Leica Microsystem tissue processor (ASP 300S, Germany). After sectioning at 3 μm, they were stained with hematoxylin and eosin (HE) and examined under a light microscope. Masson’s trichrome staining was also performed to detect fibrosis by modified masson’s trichrome stain Kit (Sigma, USA).

### Western blot analysis

Myocardial tissue was solubilized with a lysis buffer contained protease and phosphotase inhibitors to prepare protein extracts. For western blotting, equal amount of proteins were resolved on sodium dodecyl sulfate polyacrylamide gel electrophoresis and bands then transferred to polyvinylidene difluoride membranes. The membranes were blocked with blocking buffer (5% nonfat milk in tris-buffered saline (TBS-T)) for 1 h, then incubated overnight at 4 °C with the appropriate primary antibodies (Abcam, UK). After washed by TBS-T, blot was incubated with the secondary antibodies (Abcam, UK) for 2 h at room temperature. After immunoblotting, stained bands were detected by enhanced chemiluminescence using the ECL detection kit (Bio-Rad, USA). Glyceraldehyde-3-phosphate dehydrogenase (GAPDH) (Abcam, UK) was used as the loading control.

### Real-time quantitative PCR analysis

Total RNA was extracted and reverse-transcribed by TRIzol (Takara Biotechnology, Tokyo, Japan) and complementary DNA reverse transcription kit (Invitrogen, USA), respectively. The real-time PCR reaction was performed using the real-time PCR thermocycler (Bio-Rad, USA). The comparative threshold cycle method was used to calculate the mRNA expression of the target genes normalized to GAPDH. The primer sequences were as follows: SOD-1 5′-GAACCATCCACTTCGAGCAG-3′ (forward), 5′-GATGGACGTGGAACCCATGC-3′ (reverse); SOD-2 5′-CGGCCTACGTGAACAATCTC-3′ (forward), 5′-TTAGGGCTCAGGTTTGTCCAG-3′ (reverse); Bax 5′-CTCCGGCGAATTGGAGATGAA-3′ (forward), 5′-CAGTTGAAGTTGCCATCAGC-3′ (reverse); Bad 5′-GAGCAGGAAGACGCTAGTGC-3′ (forward), 5′-GGTACGAACTGTGGCGACTC-3′ (reverse); Bcl-2 5′-TGAGTACCTGAACCGGCATC-3′ (forward), 5′-AAGCCCAGACTCATTCAACCA-3′ (reverse); Nrf2 5′-CCCAGCAGGACATGGATTTGA-3′ (forward), 5′-AGCTCATAGTCCTTCTGTCGC-3′ (reverse); Keap1 5′-CCAGATTGACAGCGTGGTTC-3′ (forward), 5′-GGTTGAAGAACTCCTCCTGCT-3′ (reverse); GAPDH 5′-GGTCCCAGCTTAGGTTCATCA-3′ (forward), 5′-CCGTTCACACCGACCTTCA-3′ (reverse).

### Data and statistical analysis

All values are expressed as means ± standard deviation (SD). Statistical significance was defined as a two-sided *P* value < 0.05. One-way analysis of variance (ANOVA), followed by LSD-t test, was used to compare each variable for differences among the groups, using SPSS 20.0 software (IBM, USA).

## Results

### Cold exposure induced cardiac injury

**Fig. 1:**
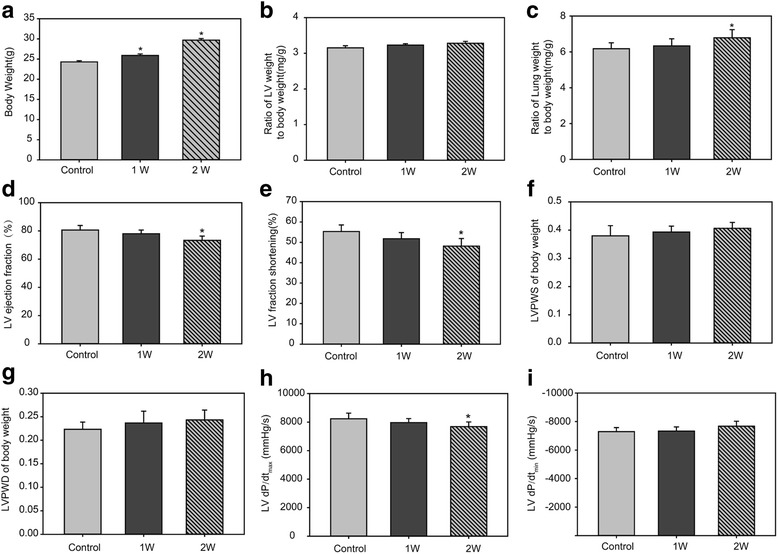
Physiological changes of the mice under cold exposure. **a** Body weight. **b** Ratio of LV weight to body weight. **c**: Ratio of lung weight to body weight. **d** LV ejection fraction. **e** LV fraction shortening. **f** LVPWS of body weight. **g** LVPWD of body weight. **h** LV dP/dt_max_. (**i**) LV dP/dt_min_. Data are mean ± SD. * *p* < 0.05, compared to control group

Under cold exposure, body weight was increased with exposure time, so the 1 w and 2 w cold exposure groups were heavier than the control groups (*P* < 0.05, Fig. 1a). Ratio of left ventricular weight to body weight in the two experimental groups were also a little higher than in the control group (Fig. 1b), but not significantly, indicating that cold stress maybe exacerbated myocardial hypertrophy slightly. Cold stress also led to increased ratios of lung weight to body weight, suggesting that LV dysfunction occurred after cold exposure for 2 weeks (Fig. 1c).

Echocardiography showed that cold exposure decreases in LV ejection fraction and LV fractional shortening (Fig. 1d and e). And the left ventricular posterior wall thickness at end systole (LVPWS) and diastole (LVPWD) of the body weight showed the opposite trend, but not significantly (Fig. 1f and g). LV dP/dt_max_ was decreased under cold exposure, further demonstrating LV dysfunction in the experimental groups (Fig. 1h). However, LV dP/dt_min_ was just a little increased (Fig. 1i).

**Fig. 2:**
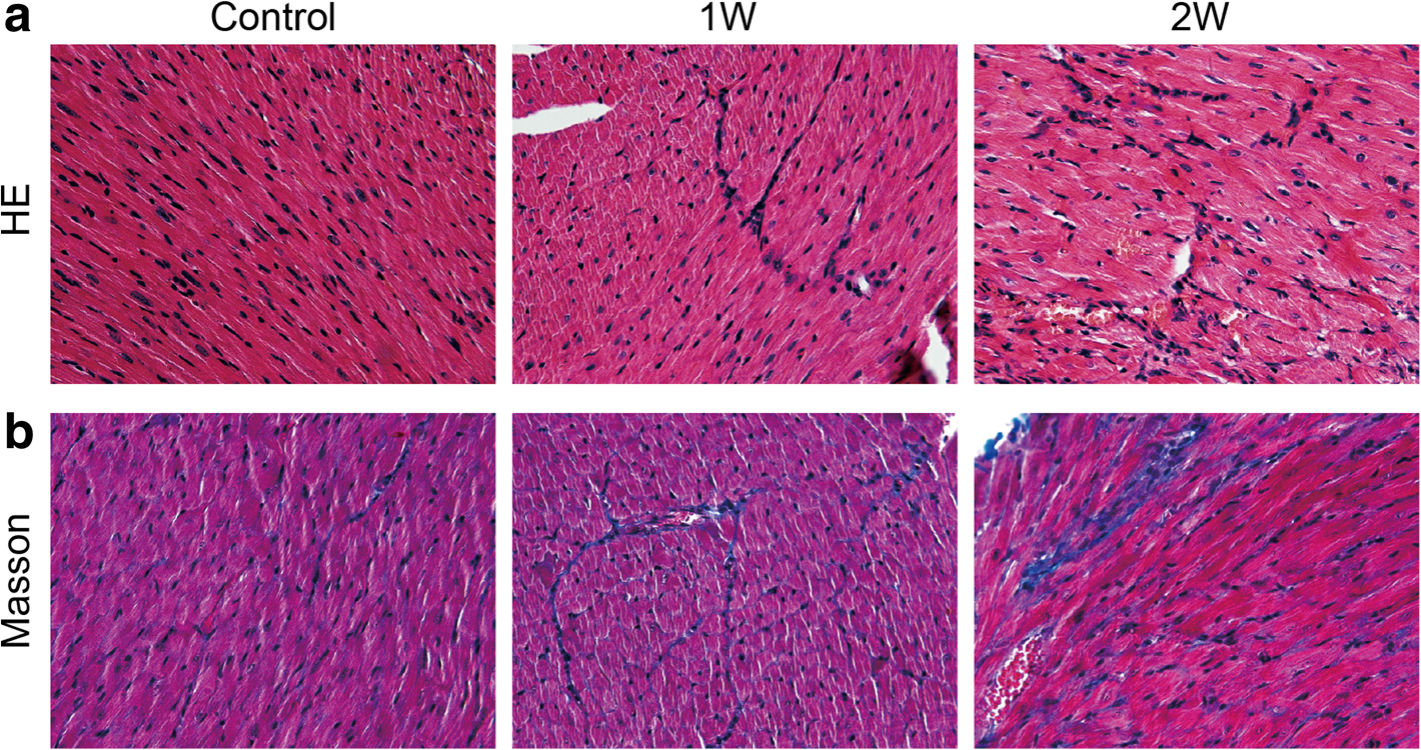
Histopathological changes of myocardium under cold exposure. **a** HE staining of myocardium in different groups. **b** Masson stained sections of left ventricles in different groups

Histological staining of LV tissues showed that inflammation and vacuolar degeneration occurred in cardiac myocyte in the 1-week cold exposed mice. And eosinophilic degeneration was witnessed in the 2-week cold exposed mice (Fig. 2a). There were more blue stained area in the 2-week cold exposed mice, which demonstrated that myocardial fibrosis was observed by Masson’s trichrome staining (Fig. 2b).

### Cold exposure induced cardiac oxidative stress

**Fig. 3:**
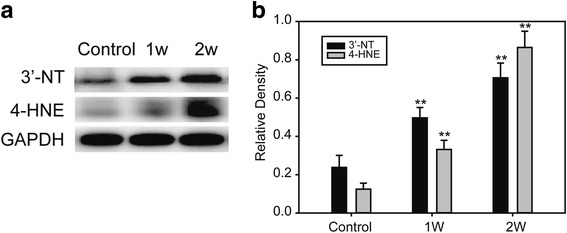
Oxidative stress markers in myocardium under cold exposure. **a** Western blot of 3’-NT and 4-HNE. **b** Relative density of 3’-NT and 4-HNE. Data are mean ± SD. * *p* < 0.05, compared to control group, ** *p* < 0.01, compared to control group

To examine the potential mechanism of action of cold exposure-induced myocardial contractile dysfunction, oxidation markers were examined in the myocardium. The protein and lipid oxidation markers 3′-nitrotyrosine (3’-NT) and 4-hydroxynonenal (4-HNE) were measured to assess effects of cold stress on myocardial oxidative stress. Western blotting for 3′-NT and 4-HNE showed that both increased under cold exposure (Fig. 3a and b), indicating oxidative stress in the myocardium.

### Cold exposure affected myocardial antioxidant enzymes

**Fig. 4:**
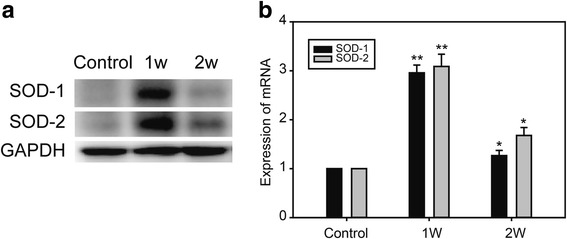
Antioxidant enzymes changes in myocardium under cold exposure. **a** Western blot of SOD-1 and SOD-2. **b** Real time PCR of SOD-1 and SOD-2. Data are mean ± SD. * *p* < 0.05, compared to control group, ** *p* < 0.01, compared to control group

To assess effects on antioxidant enzymes during oxidative stress in the myocardium under cold exposure, protein and mRNA levels of these enzymes were measured in tissue from the LV myocardium. Western blotting results demonstrated that superoxide dismutase (SOD)-1 and SOD-2 levels were increased in 1-week cold exposed and decreased in 2-week cold exposed mice (Fig. 4a). Expression of SOD-1 and SOD-2 mRNA in the LV myocardium, detected by real-time RT-PCR, also indicated the same changes as observed with the proteins (Fig. 4b).

### Cold exposure increased apoptosis in the myocardium

**Fig. 5:**
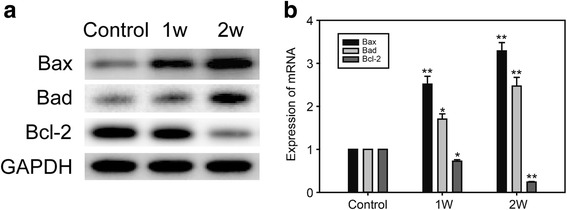
Pro-apoptotic proteins in myocardium under cold exposure. **a** Western blot of Bax, Bad and Bcl-2. **b** Real time PCR of Bax, Bad and Bcl-2. Data are mean ± SD. * *p* < 0.05, compared to control group, ** *p* < 0.01, compared to control group

Apoptosis can be triggered as a result of oxidation. So we examined expression of pro-apoptotic proteins and anti-apoptotic protein in myocardium under cold exposure. Expression of Bax and Bad increased in 1-week and 2-week cold exposed mice, and Bcl-2 decreased in two experimental groups (Fig. 5a). And expression of mRNA in Bax and Bad was significantly elevated in the cold exposed groups, which opposite to expression of mRNA in Bcl-2 (Fig. 5b).

### Cold exposure induced oxidative stress and apoptotic injury by inhibiting the Nrf2-Keap1 signaling pathway

**Fig. 6:**
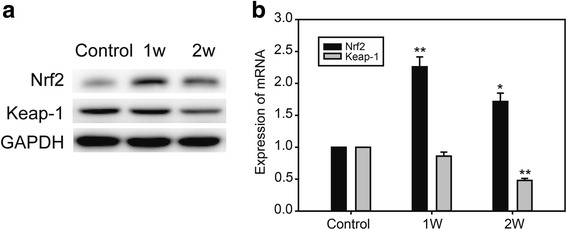
Pro-apoptotic proteins in myocardium under cold exposure. **a** Western blot of Nrf2 and Keap-1. **b** Real time PCR of Nrf2 and Keap-1. Data are mean ± SD. * *p* < 0.05, compared to control group, ** *p* < 0.01, compared to control group

In our study, western blotting showed that levels of the cellular sensor protein for oxidative stress, Nrf2, were increased in the 1-week, then decreased in the 2-week, exposure group. Levels of Keap1, a promoter of Nrf2 degradation by autophagy, were decreased under cold exposure (Fig. 6a). The same trends were observed measuring mRNA levels of the two genes (Fig. 6b).

## Discussion

The major findings of our study were that cold exposure induced: (i) an oxidative stress response, indicated by subsequent effects on myocardial antioxidant enzymes; (ii) apoptosis in the myocardium; and (iii) inhibition of the Nrf2-Keap1 signaling pathway by decreasing Nrf2 expression. These findings implied that cold stress induced oxidative stress, apoptosis and LV dysfunction by regulating the Nrf2-Keap1 signaling pathway.

In recent years, several reports were focus on chronic low temperature exposure induced myocardial dysfunction, and witnessed cardiac hypertrophy and fibrosis by intracellular Ca2+ derangement, oxidative stress, mitochondrial damage and apoptosis [20–22]. But extreme cold weather induced cardiac injury was seldom reported. In this study, we tried to explore the changes in myocardium under extreme cold weather and found followings. Increased ratios of lung weight to body weight, decreased LV ejection fraction and LV fractional shortening to body weight and decreased LV dP/dt_max_ were witnessed in the 2-week cold exposure group of this study (Fig.1). All these physiological changes demonstrated LV dysfunction induced by cold exposure. Histological analyses witnessed inflammation, vacuolar degeneration, eosinophilic degeneration and fibrosis occurred in the myocardium (Fig.2). As a result, they further demonstrated that 2-week cold exposure could lead to cardiac injury.

Injury induced by myocardial oxidative stress is determined by the balance between oxydate and their elimination by various antioxidants in the tissue. In recent studies, antioxidant defense enzymes exhibited a compensatory increase following long-term cold exposure, probably indicating an important role of oxidative stress in cold-related health problems [21, 23]. Oxidative metabolism in the mitochondria is the major source of myocardial ATP. Similarly, the primary site for reactive oxygen species (ROS) generation in cardiomyocytes is the mitochondria [24, 25]. 4-HNE is a α, β-unsaturated hydroxyalkenal, generated by peroxidation of n-6 polyunsaturated fatty acids. High 4-HNE levels, under conditions of high oxidative stress, can induce a variety of biological responses, such as cellular dysfunction and apoptosis [26–31]. We observed increased levels of 3’-NT and 4-HNE, two widely used markers for myocardial oxidative stress. Their levels increased with length of cold exposure (Fig 3).

As a member of the bZIP transcription factor family, Nrf2 plays a well-established role against oxidative stress. Nrf2 remains at low levels under normal conditions because of its rapid ubiquitylation and proteasome dependent degradation. During oxidative stress, Nrf2 degradation was inhibited in various organs, such as the heart, brain, kidney, pancreas and liver [32–39]. Keap1 modification by HNE enables release and nuclear translocation of Nrf2, and this process is possibly modulated by mitochondrial ROS production. Oxidative stress led to oxidation of critical cysteine residues in Keap1, disrupting the Keap1 ubiquitination system [40]. It was confirmed that Nrf2/Keap1 signaling pathway could be activated to regulate protective effect against cardiovascular disease [41]. In our study, Nrf2 levels in the nucleus increased in the 1-week and decreased in the 2-week cold exposed group. Keap1 levels followed a downward trend with longer cold exposure, suggested that prolonged cold stress would impede transcriptional activation by Nrf2. Higher Bax expression with longer cold stress exposure was further evidence for apoptosis, likely triggered by the oxidative stress (Fig. 5). These results were different from those in other reports [38–40] and we propose two possible reasons for the discrepancy. One is that Nrf2 levels were exhausted under long-term stress. Two-week’s continuous stimulation was given in our study, which opposite to short-term stress was given in several Nrf2-related studies [41–43]. Moreover, it was suggested that persistent accumulation of Nrf2 in the nucleus has toxic effects, such as inducing apoptosis and aggravating oxidative stress [44, 45]. So such effects might have caused negative feedback, leading to decreased Nrf2 in the 2-week cold exposed group.

Various mitochondrial antioxidant enzymes, including manganese SOD-2, are important defenses against mitochondrial ROS. In many studies, these mitochondrial antioxidant enzymes protected the myocardium against oxidative injury and ventricular dysfunction under oxidative stress [46–51]. Such protection was also a result of Nrf2 activation [52–54]. Our findings showed that levels of SOD enzymes were increased in 1-week and decreased in 2-week cold exposed groups, likely because of Nrf2 levels (Figs. 4 and 6).

Our results implied that the cold exposure first triggered oxidative stress, leading to active 4-HNE-dependent modifications in the myocardium. At the same time, the Nrf2 signaling pathway was activated and oxidative stress disrupted the critical cysteine residues in Keap1, enabling Nrf2 to translocate into the nucleus from the cytoplasm. Progressively, Nrf2 exerted its anti-oxidative effects, activating transcription of antioxidant enzymes, including SOD-1 and SOD-2. However, with longer cold exposure, Nrf2 levels were suppressed, leading to decreased levels of antioxidant enzymes, enabling aggravation of oxidative stress and apoptosis.

## Conclusion

In summary, we demonstrated that cold exposure induced cardiac injury by inhibited the Nrf2-Keap1 signaling pathway. Our data indicated that apoptosis, 4-HNE production, decreased Nrf2 levels and the resulting cardiac injury may be essential to the adverse effects of cold exposure. Given the role of antioxidants in protection against cardiac injury, our data supported the therapeutic potential of antioxidants for managing cold stress-associated myocardial complications.

## Abbreviations

3’-NT: 3′-nitrotyrosine
4-HNE: 4-hydroxynonenal
Cul3: Cullin 3
GAPDH: Glyceraldehyde-3-phosphate dehydrogenase
HE: Hematoxylin and eosin
Keap1: Kelch like-ECH-associated protein 1
LV: Left ventricle
LVPWD: Left ventricular posterior wall thickness at end diastole
LVPWS: Left ventricular posterior wall thickness at end systole
Nrf2: Nuclear factor erythroid-derived 2-like 2
SOD: Superoxide dismutase
TBS-T: Tris-buffered saline
